# Autophagic Degradation Deficit Involved in Sevoflurane-Induced Amyloid Pathology and Spatial Learning Impairment in APP/PS1 Transgenic Mice

**DOI:** 10.3389/fncel.2018.00185

**Published:** 2018-07-03

**Authors:** Pengcheng Geng, Jiqian Zhang, Wei Dai, Xiaoyu Han, Qilian Tan, Dan Cheng, Panpan Fang, Xuesheng Liu

**Affiliations:** Department of Anesthesiology, First Affiliated Hospital of Anhui Medical University, Hefei, China

**Keywords:** Alzheimer’s disease, cognitive dysfunctions, sevoflurane, autophagic degradation, enlarged autolysosomes

## Abstract

The adverse effects of anesthetics on elderly people, especially those with brain diseases are very concerning. Whether inhaled anesthetics have adverse effects on Alzheimer’s disease (AD), which is the most common form of dementia with brain degenerative changes, remains controversial. Autophagy, a crucial biological degradation process, is extremely important for the pathogenesis of AD. In this study, the inhaled anesthetic sevoflurane elicited many enlarged autolysosomes and impaired the overall autophagic degradation in the hippocampus of an AD mouse model, which is involved in the accumulation of amyloid-β (Aβ) and spatial learning deficits. However, rapamycin treatment counteracted all these effects. The results suggested that inhaled anesthetics may accelerate the pathological process of AD, and enlarged autolysosomes may be a new marker for prediction and diagnosis of the neurotoxicity of anesthetics in AD.

## Introduction

Alzheimer’s disease (AD) is the most common form of dementia with brain degenerative changes, which is highly prevalent in the elderly population (Querfurth and LaFerla, 2010). With the global increase in elderly population, more AD patients will receive surgical treatment and general anesthesia. Due to its advantages of rapid onset, strong controllability, and fewer side effects, inhaled anesthesia has become the most common method of general anesthesia. However, growing evidence revealed that the aged brain is vulnerable to inhaled anesthetics (Vutskits and Xie, 2016), for example, isoflurane, sevoflurane and desflurane can impair the cognitive function of older rodents (Culley et al., 2003; Le Freche et al., 2012; Li et al., 2014; Callaway et al., 2015). Although the effects of inhaled anesthetics on old animals are widely reported, that on AD population is rare. Several studies have demonstrated that inhaled anesthetic isoflurane could impair spatial learning of AD mouse model (Li et al., 2014), while other studies have shown that isoflurane and desflurane have no effect on or promote spatial learning and memory in AD mouse model (Sturchler-Pierrat et al., 1997; Oddo et al., 2003; Bianchi et al., 2008). Thus, the effect of inhaled anesthetics on cognitive function of AD animal models remains controversial.

Numerous laboratory and clinical studies have revealed some AD-associated pathological changes in inhaled anesthetic-exposed elderly brain. Isoflurane and other inhaled anesthetics could increase the levels of β-secretase and γ-secretase that are involved in amyloid-β (Aβ) generation (Tanzi and Bertram, 2005) and further induce dose and exposure duration-dependent Aβ accumulation and apoptosis (Xie et al., 2007; Dong et al., 2009). In addition, inhaled anesthetics could also promote the hyperphosphorylation of tau, which is another pathognomonic feature of AD (Le Freche et al., 2012; Li et al., 2014). Similar observations have been made in humans wherein elevated levels of tau and Aβ1–42 were found 1 week after surgery in the cerebrospinal fluid (CSF) of patients who underwent coronary artery bypass graft under general anesthesia (Palotás et al., 2010). Progressive increase in both total and phosphorylated tau levels in the CSF during the first 48 h post-operation have also been reported in patients undergoing idiopathic nasal CSF leak correction (Tang et al., 2011). Increased CSF Aβ1–40 levels were detected 24 h after lower-extremity or lower abdominal surgery in patients under spinal anesthesia plus isoflurane (Zhang et al., 2013).

In the above pathological changes, Aβ received particular attention. Imbalance in production and degradation leads to an increase in Aβ levels, which may result in cognitive dysfunction (LaFerla et al., 2007). As the main pathway for Aβ degradation, autophagy is a lysosome-based, evolutionarily conserved and dynamic intracellular catabolic process, in which cytoplasmic constituents like Aβ are engulfed by autophagosomes and delivered to lysosomes for degradation (Xie and Klionsky, 2007). The autolysosome is the organelle that degrades autophagic cargos. However, emerging evidence suggested that autophagy flux in late stage of AD is impaired owing to autolysosomal/lysosomal dysfunction (Bordi et al., 2016), but reversal autophagy degradative function can ameliorate amyloid pathologies and memory deficits in the AD mouse model (Yang et al., 2011). Accordingly, whether inhaled anesthetic impairs autophagy of AD, induces Aβ accumulation and impairs cognitive function remains unknown.

Sevoflurane is the most widely used halogenated inhaled anesthetic. However, increasing evidence has shown that sevoflurane may induce AD-like pathological changes and cognitive dysfunction in some cases (Xie et al., 2006; Liu et al., 2010). In this study, we found that sevoflurane-induced enlarged autolysosomes might be a new marker for predicting the neurotoxicity of anesthetics in AD. Additionally, sevoflurane disrupted the autophagic degradation and led to Aβ accumulation, which might cause spatial learning deficits in APP/PS1 mouse. However, rapamycin treatment could abrogate the enlarged autolysosomes, prevent Aβ degradation, and improve mouse spatial learning. Thus, rapamycin may be a promising candidate to prevent inhaled anesthetic-induced cognitive impairment in AD patients.

## Materials and Methods

### Animals

Six to seven-month-old male double transgenic APPswe/PSEN1dE9 mice (known as APP/PS1) were obtained from the Model Animal Research Center of Nanjing University. All animals were housed in temperature, humidity and light-controlled rooms, with food and water provided *ad libitum*. Animals used in this study were obtained from the Model Animal Research Center of Nanjing University. Animal welfare and experimental procedures were carried out in accordance with the Ethical Regulations on the Care and Use of Laboratory Animals of Anhui Medical University and were approved by the school committee for animal experiments.

### Treatment and Anesthesia

The APP/PS1 mouse were randomly assigned to control groups, sevoflurane group, rapamycin group and sevoflurane plus rapamycin group. Rapamycin (Shanghai Macklin Biochemical, R817296) stock solution was prepared in ethanol, stored frozen at 25 mg/ml, and then diluted 10-fold with vehicle (5% Tween-80 and 5% polyethylene glycol 400 in double deionized water) immediately before use. Ethanol was also added to the vehicle solution. Control and sevoflurane group received intraperitoneal injections of vehicle while rapamycin and sevoflurane plus rapamycin group received rapamycin at 15 mg/kg/day three times a week for 3 weeks (Siman et al., 2015). Twenty-four hours after the last treatment, the sevoflurane and sevoflurane plus rapamycin group received 3% sevoflurane plus 60% oxygen (balanced with nitrogen) for 4 h in a thermostated chamber, while the control and rapamycin group received 60% oxygen only. We monitored the rectal temperature of mice (Wi78653, Dongxi Instrument) and controlled the anesthesia chamber temperature at 37 ± 1°C by placing a warming pad under the chamber. The mice breathed spontaneously, and the concentrations of the anesthetic and oxygen were measured continuously using a calibrated Datex (Ohmeda, GE Healthcare, Tewksbury, MA, USA). Arterial blood samples were collected in capillary tubes and analyzed for pH, PaCO_2_ and PaO_2_, (ABL5, Radiometer Medical A/S, Bronshoj, Denmark). These mice were used only to determine the physiologic parameters during anesthesia.

### Immunofluorescence

To obtain tissues for experiments, animals were anesthetized by i.p. injection of 1% pentobarbital.

One hemisphere was frozen at –80°C and the other half was immersion-fixed in 4% paraformaldehyde at 4°C overnight (Yang et al., 2011). The brain was dehydrated in 30% sucrose, cryoprotected in OCT, and sectioned at 8 μm thickness in the coronal plane (Bregma −2.2 ± 0.5 mm) on a freezing microtome (SLEE, Germany). Slices were washed with TBST, blocked with 4% bovine serum albumin (BSA), and incubated with primary antibodies (Aβ; 1:200, Abcam, lysosomal-associated membrane protein 1, LAMP1; 1:500, Abcam and light chain 3, LC3; 1:100, Novus Biologicals) overnight at 4°C. After washing with wash buffer containing 1% Tween 20, the slices were incubated with the secondary antibodies (Alexa Fluor 568; 1:500, Invitrogen, Alexa Fluor 488; 1:500, Invitrogen and Alexa Fluor 647; 1:500, Invitrogen) at 37°C for 1 h. The sections were examined under a Zeiss LSM710 confocal or Olympus IX71 fluorescence microscope and analyzed by ImageJ software. The lysosomal-related organelles and autolysosomes which positively stained by LAMP1 and CathD with more than 1 μm of diameter were defined as enlarged autolysosomes. Cells with more than three of these structures were counted as cells with enlarged autolysosomes (Zhang et al., 2017). The size of LAMP1 and CathD structures, number and area of amyloid plaques were measured by Image J software using its “analyze particle” analysis tool with default image/adjust/threshold settings.

### Western Blotting Analysis

The hippocampus was homogenized in PBS followed by RIPA buffer (50 mM Tris-HCl, 150 mM NaCl, 1% Triton X-100, 0.1% SDS, and 1X protease inhibitor. The total protein concentration was determined using a BCA kit (Beyotime Biotechnology, China). Total protein extracts were normalized to 1 μg/μl, boiled for 10 min, separated by electrophoresis on an SDS-polyacrylamide gel, and transferred to a polyvinylidene fluoride (PVDF) membrane (Millipore, Germany). After blocking with 5% nonfat dry milk for 1 h, the PVDF membrane was incubated with primary antibodies (LC3; 1:2000, Novus Biologicals, Aβ; 1:1000, Abcam, APP; 1:1000, Abcam, Cathepsin D (CathD); 1:1000, Santa Cruz, p62; 1:1000, Abcam, β-actin; 1:1000, Transgene) at 4°C overnight, extensively washed, incubated with a horseradish peroxidase-conjugated secondary antibody (1:10,000, Promega) for 1 h, and then visualized with an ECL kit.

### Enzyme-Linked Immunosorbent Assay

The hippocampus was homogenized in PBS followed by RIPA buffer (50 mM Tris-HCl, 150 mM NaCl, 1% Triton X-100, 0.1% SDS, and 1X protease inhibitor (Xiao et al., 2014). The concentrations of soluble Aβ42 and Aβ40 were then detected in the supernatant by a human specific ELISA kit (CUSABIO, China) according to the manufacturer’s instructions.

### Morris Water Maze (MWM)

Forty mice (*n* = 10/group) were used in this test. To evaluate the spatial learning, mice were trained in a circular water maze (1.2 m in diameter, opaque water, 50 cm deep, 19–21°C) with a platform (10 cm in diameter) hidden 1-cm below water 1 week after exposure. The pool was surrounded by a black curtain and was located in an isolated room with four visual cues on the wall of pool. The form and color of the visual cues were different and the size of visual cues were about 12 cm in diameter. Mice were given four trials per day in the MWM for four consecutive days. The start position was pseudorandomized across trials. A trial was terminated when the animal reached the platform, where it was allowed to remain for 30 s. If the animal failed to find the target before 90 s, it was manually guided to the platform, where it was allowed to stay for 30 s. After each trial, the mice were placed in a holding cage under a heat lamp for 1–2 min to dry. The escape latency was recorded by a video tracking system. On the fifth day, the platform was removed, and the mouse was placed in the opposite quadrant. Each mouse was allowed to swim in the pool for 90 s, and the platform crossing times and swimming speed were recorded. The animals used in behavioral assays are different from those used for molecular biological testing.

### Statistical Analysis

SPSS (version 19.0) was used for statistical analysis, and the data were expressed as the mean ± SD. Differences among groups were analyzed one-way ANOVA followed by Tukey’s *Post-hoc* test or unpaired two-tailed Student’s *t*-test. For the hidden-platform training of the MWM test, mean escape latency of mice was analyzed by two-way repeated-measures ANOVA followed by Tukey *post hoc* test. Mann–Whitney U-test was used to compare the platform crossing times of mice. Differences were considered statistically significant at *p < 0.05 and **p < 0.01, ***p < 0.001.

## Results

### Sevoflurane Induces Aβ Accumulation in the Hippocampus

**Figure 1:**
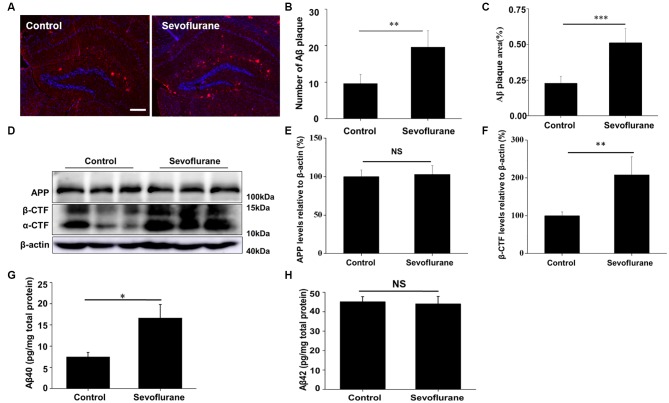
Sevoflurane induces amyloid-β (Aβ) accumulation. Six to seven-month-old APP/PS1 mice were continuously exposed to 3% sevoflurane or 60% oxygen for 4 h (*n* = 5/group). **(A)** Seven days after sevoflurane exposure, brain sections were immunostained with the anti-Aβ antibody, and the nuclei were stained with Hoechst. Scale bar = 500 μm. **(B,C)** Statistical results of the number and relative area of Aβ plaques per field. **(D)** Western blotting results of the APP, α-CTF, β-CTF and β-actin of the mouse hippocampus 24 h after sevoflurane exposure. **(E,F)** Quantification of the ratio of APP and β-CTF to β-actin. **(G,H)** Soluble Aβ40 and Aβ42 levels were detected by ELISA, 24 h after sevoflurane exposure. NS, no significant difference. Data are presented as Mean ± SD, *p < 0.05, **p < 0.01, ***p < 0.001.

The blood gases were analyzed and no differences were found in pH, PaCO_2_ and PaO_2_ (Supplementary Table S1). We also detected the Aβ levels in the hippocampus of APP/PS1 transgenic mice (Kapila et al., 2014). As shown in Figures 1A–C, sevoflurane markedly increased the number (*t*_(8)_ = 3.1, *p* < 0.01) and area of Aβ plaques (*t*_(8)_ = 2.9, *p* < 0.01) in the hippocampus by 1.9 and 1.6-fold, respectively, after 7 days. Amyloid plaques are closely related to Aβ pools (Oddo et al., 2006), and hippocampal β-carboxy-terminal fragment (β-CTF) levels were found to be significantly higher (*t*_(8)_ = 3.6, *p* < 0.01) in the sevoflurane-treated mice (Figures 1D–F). Soluble Aβ is much more neurotoxic than its insoluble forms (Peric and Annaert, 2015). Our results showed that the levels of soluble Aβ40 were significantly higher (*t*_(8)_ = 2.7, *p* = 0.02) in the sevoflurane-treated mice (Figure 1G). However, soluble Aβ42 levels were not obviously different between the groups (*t*_(8)_ = −0.2, *p* = 0.82), perhaps because of an increase in the insoluble form (Figure 1H). Taken together, these data demonstrated that sevoflurane induced Aβ accumulation in the mouse hippocampus.

### Sevoflurane Induces Autophagosome Formation in the CA1 Region of Hippocampus

**Figure 2:**
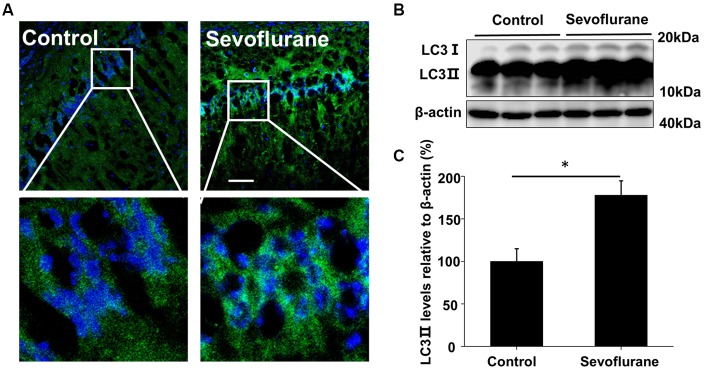
Sevoflurane induces autophagosome formation in the hippocampus. APP/PS1 mice were continuously exposed to 3% sevoflurane or 60% oxygen for 4 h (*n* = 5/group); 24 h after sevoflurane exposure, autophagy induction was detected. **(A)** Immunofluorescent staining for the autophagosome marker LC3 (green) and Hoechst-stained nuclei in hippocampal sections. Scale bars = 100 μm. **(B)** Western blotting results of LC3 and β-actin levels in the mouse hippocampus. **(C)** Quantification of the ratio of LC3 ∥ to β-actin. Data are presented as Mean ± SD, *p < 0.05.

The production of Aβ is a continuous dynamic process. APP cleavage and the subsequent Aβ degradation simultaneously determine the Aβ levels in cells (Nilsson et al., 2013). Accordingly, we detected the APP levels and there was no significant difference in the hippocampus of the control and sevoflurane-treated mice (*F*_(3,16)_ = 0.4, *p* = 0.74; Figure 1D). As autophagy is the main pathway for Aβ degradation, we tested the induction of autophagy in the mouse CA1 region of hippocampus. During autophagy, the autophagy marker protein LC3 is cleaved from LC3 I to a lower molecular weight LC3 II and aggregates on the autophagosome membranes (Mizushima et al., 2010). Thus, using immunofluorescence staining, we found that sevoflurane induced numerous LC3 puncta, indicating the formation of autophagosomes (Figure 2A). Furthermore, the LC3 II levels in the sevoflurane-treated mice were 1.7-fold higher (*t*_(8)_ = 6.0, *p* < 0.0.01) than that in the control mice, further confirming the formation of autophagosomes (Figures 2B,C).

### Sevoflurane Elicits Enlarged Autolysosomes and Impairs Autophagic Degradation

**Figure 3:**
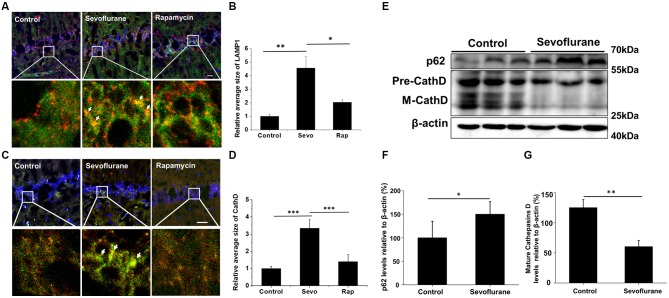
Sevoflurane induces enlarged autolysosomes and impairs autophagic degradation in the hippocampus of AD mice. APP/PS1 mice were continuously exposed to 3% sevoflurane or 60% oxygen for 4 h (*n* = 5/group). For rapamycin treatment, mice were intraperitoneally pre-injected with 15 mg/kg/day rapamycin every 2 days for 3 weeks. Twenty-four hours after sevoflurane exposure, enlarged autolysosomes were observed and autophagic degradation ability was detected. **(A,C)** Confocal images of dual immunofluorescence labeling for light chain 3 (LC3, green) and lysosomal-associated membrane protein 1 (LAMP1, red, **A**) or cathepsin D (CathD, red, **C**) in the hippocampus of mice. Enlarged autolysosomes are indicated by arrow. Scale bar = 50 μm. **(B,D)** Quantification of the relative average size of LAMP1 and CathD. **(E)** Western blotting results of p62, CathD and β-actin in the mouse hippocampus. **(F,G)** Quantification of the ratio of p62 or CathD to β-actin. Data are presented as Mean ± SD, *p < 0.05, **p < 0.01, ***p < 0.001.

Autophagosomes fuse with several lysosomes to form autolysosomes, in which the autophagic contents are degraded (Mizushima et al., 2008). LAMP1, a lysosomal membrane protein, and CathD, an internal protease, are used as lysosomal markers (Yu et al., 2010). Thus, structures with overlapped immunofluorescence staining for LAMP1/CathD and LC3 were considered autolysosomes. Enlarged autolysosomes are often observed and involved in several diseases. The hippocampus of the sevoflurane-exposed mice also generated many enlarged autolysosomes (Figures 3A,C), indicative of abnormal autophagic flux. Rapamycin is a commonly used autophagy inducer, yet the sevoflurane-induced autolysosomes were approximately 2-fold larger than those in rapamycin-treated mice (LAMP1 structure *p* = 0.037, CathD structure *p* < 0.001; Figures 3B,D). Taken together, sevoflurane can elicit enlarged autolysosomes, which may contribute to sevoflurane-induced cognitive dysfunction.

Autophagic degradation failure is a common cause of enlarged autolysosomes. Hence, we examined the autophagic degradation ability in sevoflurane-exposed mice. Sequestosome 1 (SQSTM1/p62), a protein substrate that is selectively incorporated into the autophagosomes and degraded by autophagy (Bjørkøy et al., 2005), was detected. Sevoflurane treatment significantly increased the p62 levels (*t*_(8)_ = 2.7, *p* = 0.02; Figures 3E,F), which was consistent with the trend of lysosomal alkalizer CQ-treated mouse (Supplementary Figure S2), indicating impaired autophagic degradation. Furthermore, the levels of the important lysosomal protease CathD were tested. As shown in Figures 3E,G, both precursor and mature forms of CathD levels in sevoflurane-exposed mice were lower than in control mice (*t*_(8)_ = −6.8, *p* < 0.01), suggesting that sevoflurane disrupted the function of autolysosomes. Collectively, the disruption of autolysosome function caused by sevoflurane may lead to impaired autophagic degradation.

### Rapamycin Eliminates Enlarged Autolysosomes and Reverses Autophagic Degradation

**Figure 4:**
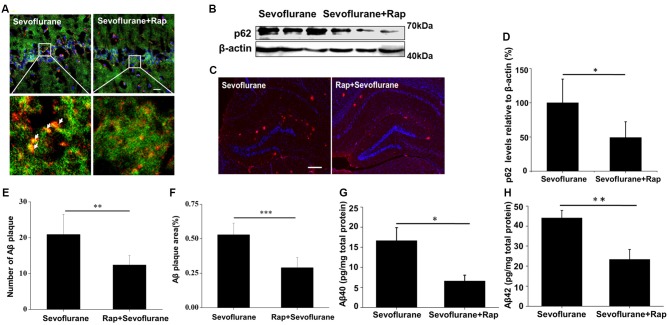
Rapamycin eliminates enlarged autolysosomes and reverses autophagic degradation. APP/PS1 mice were continuously exposed to 3% sevoflurane or 60% oxygen for 4 h (*n* = 5/group). For rapamycin treatment, mice were intraperitoneally pre-injected with 15 mg/kg/day rapamycin every 2 days for 3 weeks. **(A)** Confocal images of dual immunofluorescence labeling for LC3 (green) and LAMP1 (red) in the mouse hippocampus. Scale bar = 50 μm. **(B)** Western blotting results of p62 and β-actin in the mouse hippocampus. **(C)** Quantification of the ratio of p62 to β-actin. **(D)** Seven days after sevoflurane exposure, brain sections were immunostained with the anti-Aβ antibody and the nuclei were stained with Hoechst. Scale bars = 500 μm. **(E,F)** Statistical results of the number and relative area of Aβ plaques per field. **(G,H)** Soluble Aβ40 and Aβ42 levels were detected by ELISA, 24 h after sevoflurane exposure. Data are presented as Mean ± SD, *p < 0.05, **p < 0.01, ***p < 0.001.

Rapamycin is a novel and highly effective immunosuppressant that is clinically used for the treatment of organ transplant rejection and autoimmune diseases (Harrison et al., 2009). As a common autophagy inducer, rapamycin can trigger autophagosome formation, thereby increasing the autophagic cargo packaging efficiency and lysosomal delivery speed during autophagy. Although some autolysosomes were destroyed by sevoflurane, the higher cargo delivery efficiency induced by rapamycin might greatly improve the overall autophagic degradation. To test this hypothesis, sevoflurane-exposed mice were pre-treated with rapamycin, and no enlarged autolysosomes were observed (Figure 4A), indicating a recovery of autophagic flux. Additionally, the levels of p62 (*t*_(8)_ = −3.0, *p* = 0.01), the number (*t*_(8)_ = −2.4, *p* = 0.03) and area of Aβ plaques (*t*_(8)_ = −2.1, *p* = 0.05), and the levels of soluble Aβ40 (*t*_(8)_ = −2.9, *p* = 0.02) and Aβ42 (*t*_(8)_ = −3.3, *p* < 0.01; Figures 4B–H) were all significantly lower in the mice treated with sevoflurane plus rapamycin than in those treated with sevoflurane alone. However, rapamycin could not reverse the decline in CathD levels caused by sevoflurane (data not shown). Taken together, rapamycin improves sevoflurane-impaired overall autophagic degradation in the mouse hippocampus without reversing the impaired autolysosome function.

### Rapamycin Improves Sevoflurane-Induced Cognitive Dysfunction

**Figure 5:**
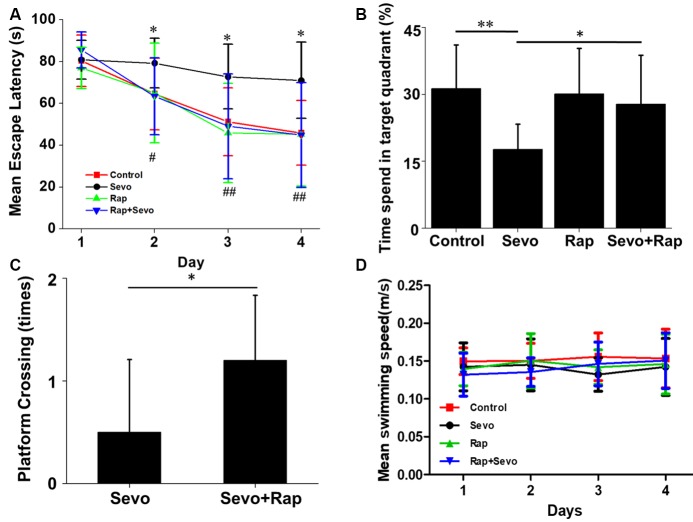
Rapamycin improves sevoflurane-induced cognitive dysfunction. APP/PS1 mice were continuously exposed to 3% sevoflurane or 60% oxygen for 4 h. For rapamycin treatment, mice were intraperitoneally pre-injected with 15 mg/kg/day rapamycin every 2 days for 3 weeks. Seven days after sevoflurane exposure, mouse spatial learning was evaluated using the Morris Water Maze (MWM) (*n* = 10/group). **(A)** Statistical results of the mean escape latency of mice. *p < 0.05, **p < 0.01, ***p < 0.001, significant differences between sevoflurane and control mice. ^#^*p* < 0.05, ^##^*p* < 0.01, ^###^*p* < 0.001, significant differences between sevoflurane and sevoflurane + rapamycin mice. **(B)** Statistical results of the percentage of times spent in target quadrant. Probe test 24 h after the last day of learning. *p < 0.05, **p < 0.01. **(C)** Statistical results of the platform crossing times. *p < 0.05. **(D)** Statistical results of the mean swimming speed. Data are presented as Mean ± SD.

**Figure 6:**
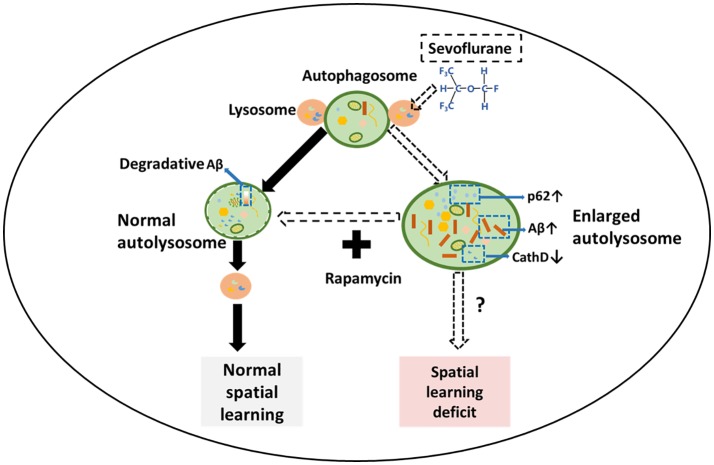
Schematic illustration of the mechanism for sevoflurane-induced cognitive dysfunction by regulating autophagy in APP/PS1 transgenic mice. In normal cells, autophagosomes fuse with several lysosomes to form autolysosomes, which are utilized to generate new lysosomes through an autophagic lysosomal reformation (ALR) process. In the sevoflurane-exposed mouse hippocampus, sevoflurane facilitates numerous enlarged autolysosomes and impairs the overall autophagic degradation, leading to the accumulation of Aβ and spatial learning. However, rapamycin treatment abrogates the enlarged autolysosomes and improves mouse spatial learning.

In AD patients, Aβ is closely related to cognitive dysfunction. Rapamycin’s ability to reduce Aβ levels suggests that it could improve sevoflurane-induced spatial learning deficit. Consistent with our hypothesis, sevoflurane increased the escape latency time of mice (*F*_(3,36)_ = 10.7, *p* < 0.01), which was prevented by rapamycin pretreatment (*F*_(3,36)_ = 6.7, *p* = 0.02; Figure 5A). Additionally, rapamycin pretreatment resulted in lower percentage of time spent in target quadrant (*F*_(3,36)_ = 3.1, *p* = 0.05) and more platform crossing times (*U* = 23.5, *p* = 0.03) after sevoflurane exposure. Notably, the only group that spends significantly different amount of time than by chance (25%) in the target quadrant was the sevoflurane group, which seems to avoid the target quadrant (Figures 5B,C). There were no significant differences in the swimming speeds between the groups (*F*_(3,36)_ = 0.05, *p* = 0.99; Figure 5D). Collectively, rapamycin can partially repair sevoflurane-induced spatial learning deficits. Hereto, the theory of this study has been schematically shown in Figure 6.

## Discussion

In the present study, the inhaled anesthetic sevoflurane led to amyloid peptide accumulation due to abnormalities in autophagy degradation machinery, which in turn caused spatial learning impairment in APP/PS1 transgenic mice. However, autophagy inducer rapamycin could counteract these effects. These results revealed that inhaled anesthetics may accelerate the pathological process of AD, reminding anesthesiologists to pay close attention to the use of inhaled anesthetics in patients with AD during clinical practice. Rapamycin may be a promising candidate to prevent anesthetic-induced neurotoxicity.

The potential neurotoxicity of anesthetics has raised increasing concerns. This study suggested that sevoflurane anesthesia could impair the spatial learning of APP/PS1 mice at 6–7 months of age, during which mice begin to generate Aβ precipitation and exhibit mild cognitive dysfunction (Holcomb et al., 1998; Wengenack et al., 2000). Consistent with our results, isoflurane administration also decreased the exploratory activity of young Tg2576 mice in the Y-maze alternation test (Perucho et al., 2010). Additionally, 4-h isoflurane exposure led to poorer spatial-memory acquisition than in wild-type APP695 mice (Games et al., 1995). However, isoflurane had no effect on cognitive function in 1-year-old adult transgenic animals, which may be due to the existing impaired cognitive performance of these animals (Bianchi et al., 2008). After isoflurane or desflurane anesthesia, no long-term behavioral effects were detected in triple transgenic mice carrying mutations in Mapt and Psen1 (Oddo et al., 2003). Contrary to our results, another study showed that learning and exploratory activity ameliorated after isoflurane exposure in the APP23 mouse strain (Sturchler-Pierrat et al., 1997). Although, there is no established consensus on the effect of inhaled anesthetics on cognitive function in animal models of AD, the different anesthesia age and onset genes may affect the experimental results.

The mechanisms underlying the neurotoxicity of inhaled anesthetics in AD are diverse, such as Aβ peptide accumulation, tau hyperphosphorylation and synaptic dysfunction, of which the Aβ pathological changes are particularly important (Vutskits and Xie, 2016). This study also found that Aβ was accumulated in sevoflurane-exposed AD mice hippocampus. Autophagy, an important biological degradative process, is the main pathway for Aβ degradation. However, this study found that sevoflurane blocked the autophagic degradation resulting in Aβ accumulation and spatial learning deficit, which was consistent with a previous study that showed autophagy was involved in sevoflurane-induced cognitive dysfunction in aged rats (Zhang et al., 2016). Notably, in our study, 3% concentration of sevoflurane is not saturated for completely blocking autophagic degradation, as lysosomal alkalizer CQ could further increase sevoflurane-induced LC3 and Aβ accumulation (Supplementary Figures S1,S2). And we choice clinically relevant concentrations of 3% aim to ensure the safety of mouse. Both degradation as well as abnormal production can lead to the accumulation of Aβ. Volatile anesthetics could lead to increase in the levels of β-secretase and γ-secretase, two proteases involved in Aβ generation (Tanzi and Bertram, 2005). Thus, APP maturation, its cleavage and enzymatic degradation should be further studied.

The degradative function of autophagy could be assayed by the levels of its substrates (Moscat and Diaz-Meco, 2009). However, hippocampal Aβ and autophagic substrate p62 levels are higher in sevoflurane-treated mice than in control mice indicating impaired autophagic degradation, which may be due to a decrease in lysosomal protease CathD. Notably, the overall autophagic degradation of sevoflurane-exposed mice recovered after rapamycin treatment, but the levels of CathD were not reversed (data not shown), suggesting persistent deficit in the autolysosomal function. Thus, we hypothesized that the overall autophagic degradation rather than the specific autolysosome function is essential to maintain Aβ levels and cognitive function of mice. However, complete autolysosomal function may also affect cognitive dysfunction in mice, since upregulating lysosomal proteases may improve cognitive function in AD mice (Yang et al., 2011). Hence, the exact relationship between autolysosomal function and cognitive function requires further study.

Postoperative cognitive dysfunction (POCD) is a common central nervous system complication characterized by a decline in cognitive performance after anesthesia and surgery (Moller et al., 1998). Concurrent POCD not only causes significant economic burden and mental stress to patients and their families but also significantly increases the mortality rate of patients. However, the mechanism of POCD remains unclear (Steinmetz et al., 2009). POCD may accelerate the pathogenesis of AD and eventually progress to AD (Vanderweyde et al., 2010; Kapila et al., 2014). General anesthetics can cause similar behavioral and pathological features in numerous animal models and POCD patients (Bianchi et al., 2008). Additionally, POCD and AD may ultimately have a common pathway. Hence, our findings in APP/PS1 transgenic mice may be crucial for understanding POCD and its mechanisms.

## Author Contributions

PG, JZ and WD designed and conducted the study, analyzed and interpreted the data and wrote the manuscript. QT, DC, XH and PF helped conduct the study. XL conceived and designed the study, analyzed and interpreted the data, drafted and critically revised the manuscript.

## Conflict of Interest Statement

The authors declare that the research was conducted in the absence of any commercial or financial relationships that could be construed as a potential conflict of interest.
