# Supplementary material for: Autophagic Degradation Deficit Involved in Sevoflurane-Induced Amyloid Pathology and Spatial Learning Impairment in APP/PS1 Transgenic Mice
Source: Front Cell Neurosci. 2018 Jul 3;12:185. doi: 10.3389/fncel.2018.00185 (PMC6037844; doi:10.3389/fncel.2018.00185)
Supplement: Supplementary file 1 [file Table_1.DOCX]

Table S1. PH, PaO_2_ and PaCO_2_ in APP/PS1 mice after 4 h of sevoflurane or oxygen exposure

| Types  Groups | PH | PaO_2_ | PaCO_2_ |
| --- | --- | --- | --- |
| Control | 7.380±0.020 | 149.75±7.36 | 39.5±2.40 |
| Sevoflurane | 7.385±0.021 | 142.25±7.91 | 41.25±2.29 |
| Rap | 7.345±0.025 | 127±6.22 | 45.25±2.56 |
| Rap+Sevoflurane | 7.348±0.034 | 129.25±5.89 | 45.75±3.07 |

n = 3 per group. Data are mean ± SEM.

pH = potential of hydrogen, PaCO_2_ = partial pressure carbon dioxide, PaO_2_ = partial pressure oxygen.
