# Supplementary material for: Autophagic Degradation Deficit Involved in Sevoflurane-Induced Amyloid Pathology and Spatial Learning Impairment in APP/PS1 Transgenic Mice
Source: Front Cell Neurosci. 2018 Jul 3;12:185. doi: 10.3389/fncel.2018.00185 (PMC6037844; doi:10.3389/fncel.2018.00185)
Supplement: Supplementary file 2 [file Presentation_1.pptx]

## Slide 1
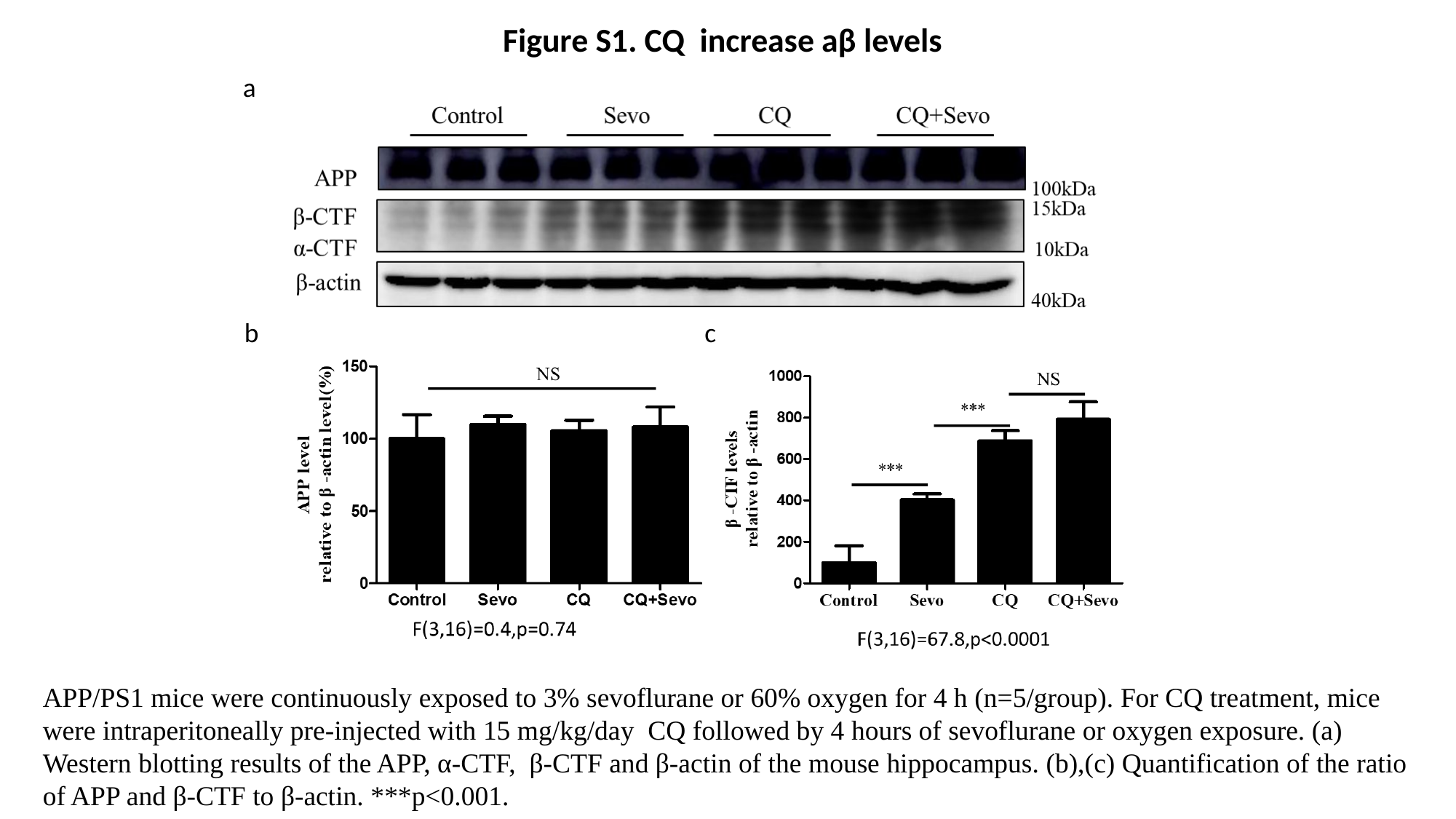

Figure S1. CQ increase aβ levels
a
b
c
APP/PS1 mice were continuously exposed to 3% sevoflurane or 60% oxygen for 4 h (n=5/group). For CQ treatment, mice were intraperitoneally pre-injected with 15 mg/kg/day CQ followed by 4 hours of sevoflurane or oxygen exposure. (a) Western blotting results of the APP, α-CTF, β-CTF and β-actin of the mouse hippocampus. (b),(c) Quantification of the ratio of APP and β-CTF to β-actin. ***p<0.001.

## Slide 2
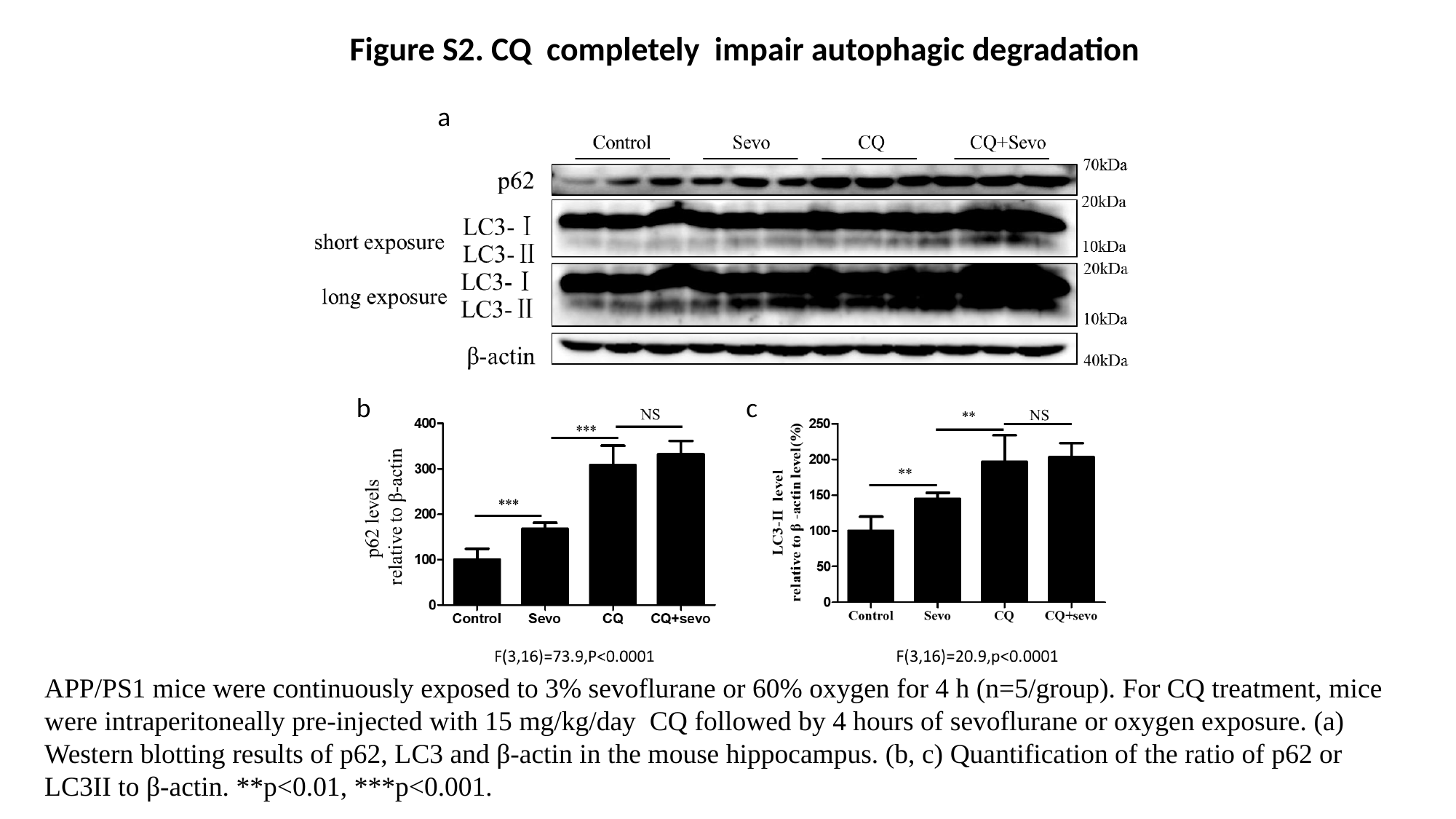

Figure S2. CQ completely impair autophagic degradation
a
b
c
APP/PS1 mice were continuously exposed to 3% sevoflurane or 60% oxygen for 4 h (n=5/group). For CQ treatment, mice were intraperitoneally pre-injected with 15 mg/kg/day CQ followed by 4 hours of sevoflurane or oxygen exposure. (a) Western blotting results of p62, LC3 and β-actin in the mouse hippocampus. (b, c) Quantification of the ratio of p62 or LC3II to β-actin. **p<0.01, ***p<0.001.
